# Modification of the contact surfaces for improving the puncture resistance of laminar structures

**DOI:** 10.1038/s41598-017-06007-3

**Published:** 2017-07-26

**Authors:** Pengfei Wang, Jinglei Yang, Xin Li, Mao Liu, Xin Zhang, Dawei Sun, Chenlu Bao, Guangfa Gao, Mohd Yazid Yahya, Songlin Xu

**Affiliations:** 1 CAS Key Laboratory of Mechanical Behavior and Design of Materials, Department of Modern Mechanics, University of Science and Technology of China, Hefei, 230027 China; 2 Department of Mechanical and Aerospace Engineering, The Hong Kong University of Science and Technology, Hong Kong SAR, China; 3 College of Mechanics, Taiyuan University of Technology, Taiyuan, Shanxi 030024 China; 4 School of Mechanical and Aerospace Engineering, Nanyang Technological University, Singapore, 639798 Singapore; 5 Department of Materials Engineering, The University of Tokyo, 7-3-1 Hongo Bunkyo-ku, Tokyo, 113-8656 Japan; 6 School of Mechanical Engineering, Nanjing University of Science and Technology, Nanjing, China; 7 Centre for Composite, Faculty of Mechanical Engineering, Universiti Teknologi Malaysia, Johor, 81310 Malaysia

## Abstract

Uncovering energy absorption and surface effects of various penetrating velocities on laminar structures is essential for designing protective structures. In this study, both quasi-static and dynamic penetration tests were systematical conducted on the front surfaces of metal sheets coated with a graphene oxide (GO) solution and other media. The addition of a GO fluid film to the front impact surface aided in increasing the penetration strength, improving the failure extension and dissipating additional energy under a wide-range of indentation velocity, from 3.33 × 10^−5^ m/s to 4.42 m/s. The coated -surfaces improved the specific energy dissipation by approximately 15~40% relative to the dry-contact configuration for both single-layer and double-layer configurations, and specific energy dissipations of double-layer configurations were 20~30% higher than those of the single-layer configurations. This treatment provides a facile strategy in changing the contact state for improving the failure load and dissipate additional energy.

## Introduction

Pangolins and snails have natural armour to defense the impact of foreign object, owing to the high strength of scales and shells. Human body armour and shields have been inspired by these biomaterials. Defense against piercing and penetrating impacts requires the protective armour with high strength and ductility for dissipating sufficient impact energy^1–3^. Various high performance nanofillers^4^ have paid more attention for their ability to improve the mechanical protection of materials. High-performance fibres can reach a high strength of 4–6 GPa, but their elongation is only 2~6%^5–8^. Aluminium alloys can withstand a relatively large plastic deformation but with a relatively low flow stress^9, 10^. To simultaneously achieve superior strength and deformable behaviour in a monomer material, combinations of these different materials in a multi-layered structure with high specific strength and energy absorption have been fabricated, but investigations of the penetration mechanisms of layered structures have been limited.

At the macroscopic scale, the fibre-epoxy laminate^11, 12^ and fibre-metal laminate (FMLs)^2, 13, 14^ composites, have been widely used in the aerospace and automobile fields due to their excellent specific strength and the diversity of available configurations. There is no doubt that a higher interfacial adhesive stress between different layers results in improving the shear energy for the lay-shear tests^15^, but there exists a critical interface cohesive stress for transverse impact loading below which the energy absorption capacity is lower than that of stacked double layers because the friction force tends to dissipate more energy^16^. Our previous findings have revealed that increasing the friction coefficient between the impactor and the structure aids in absorbing impact energies^16^. Additional friction between a projectile and a fabric prevented the mobility of the fibres and might also lead to higher energy absorption in terms of ballistic resistance^17^. The plastic deformation of materials has been found to transform from ductile to brittle and to become stronger as the compression/tension loading rate increases from the quasi-static to the dynamic regime^18, 19^. But surprisingly, the mechanical response of sandwich foam beams are similar under the quasi-static and the low-velocity impact loadings^20^, and the load-displacement curve of glass fiber metal laminates under quasi-static loading is higher than that under dynamic loading for a transverse penetration condition^21^. These results are inconsistent with the traditional velocity strengthening mechanism and require further investigation. At the microscopic level, multilayered nanocomposites have received increasing attention with the evolution of frictional characteristics^22–24^ and dynamic responses^25^. Multilayered graphene has prompted substantial research interest owing to its excellent specific energy dissipation under hypervelocity impacts^1, 26, 27^, which is related with the intrinsic strength and fracture toughness of graphene layers^28, 29^. To date, the perforation mechanisms of thin layered structures with different surface contact states have not received sufficient attention. The deformation mechanisms of surface modification and the velocity dependence of penetration in thin layered structures require additional interpretation.

Here, we present an easily performed surface embellishment methods inspired by oil or water covered skin surfaces, to improve the failure strength and absorption energy of 2D structures subjected to different indentation velocities. A graphene oxide (GO) solution -coated surface was first tested at velocities ranging over five orders of magnitude from quasi-static to dynamic penetration loadings conditions. For comparison, the pristine dry-contact aluminium layers and surfaces coated with high-vacuum grease and oil lubricant were also conducted. The specific energy dissipation showed an improvement of 15–40%, and the failure strength and the energy absorption efficiency could be redesigned while the contact surfaces were decorated. These facile surface methods elevate both the penetration strength and the deformable properties for a wide range of velocities and provide insight into the physics mechanism of different surface states in terms of the energy absorption of layered structures.

## Results

### GO-coated surface

**Figure 1:**
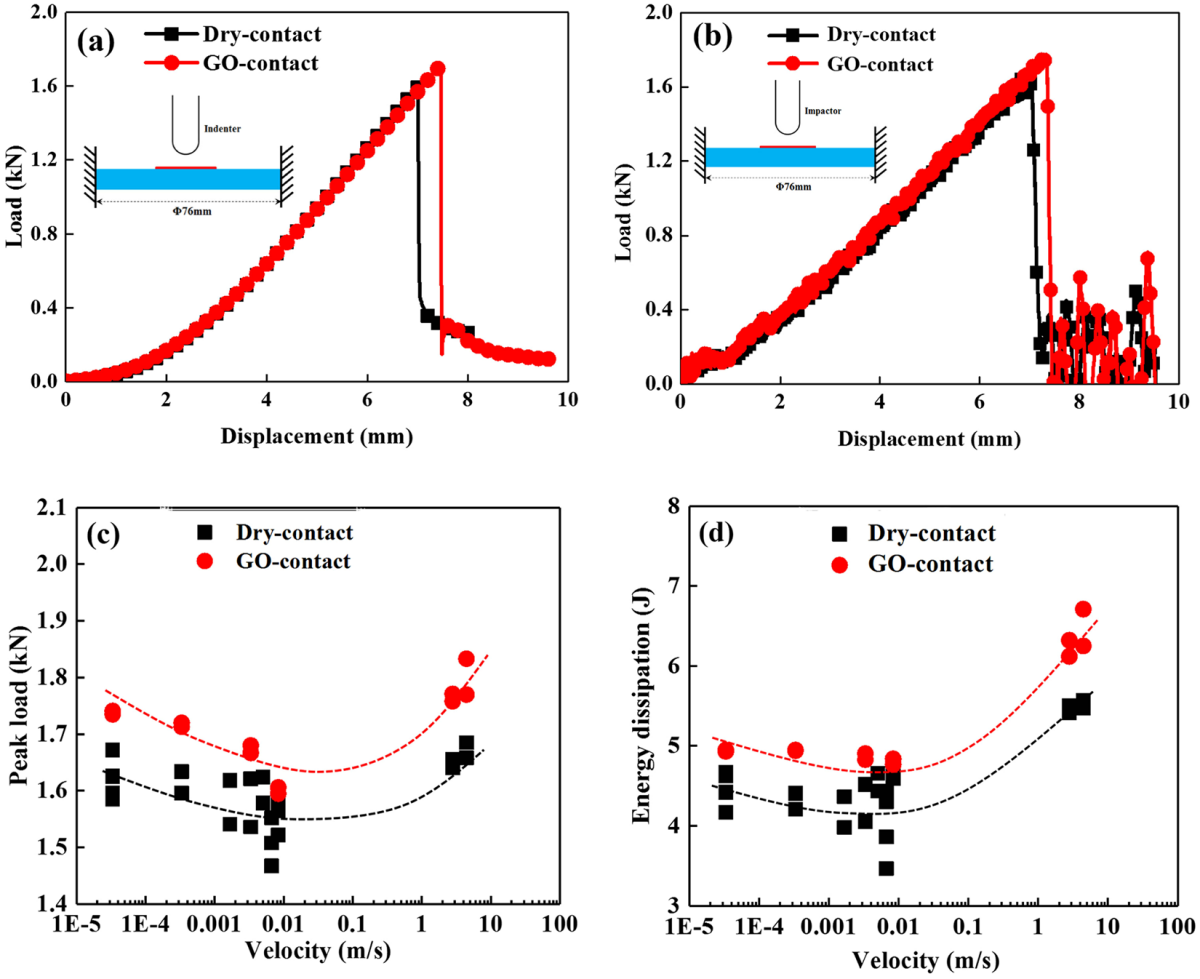
Penetration load and energy of a single-layer aluminum sheet coated with a GO solution: (**a**) load-displacement curves under 3.33 × 10^−4^ m/s (quasi-static) loading, (**b**) load-displacement curves under 2.79 m/s impact loading, (**c**) peak-load and (**d**) energy dissipation as a function of indentation velocity.

**Figure 2:**
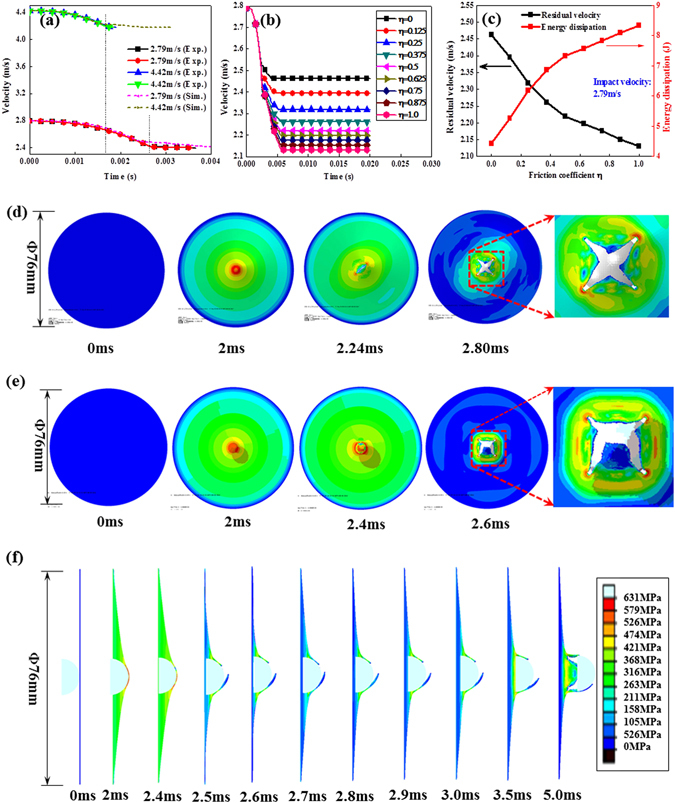
Simulation results of the penetration process of single-layer configuration: (**a**) comparison between the experimental and simulation results for contact friction coefficient η = 0.5, (**b**) simulation results of velocity-time curve under 2.79 m/s impact loading as the contact friction coefficient η increased, (**c**) simulation results of residual velocity and energy dissipation as a function of contact friction coefficient η under 2.79 m/s impact loading, (**d**) rear view of the penetration process under 2.79 m/s impact (η = 0), (**e**) rear view of the penetration process under 2.79 m/s impact (η = 0.5), and (**f**) cross-sectional view of the penetration process under 2.79 m/s impact (η = 0.5).

A thin layer of GO solution was “smeared” on the contact surface with a thickness of 10–15 μm. Typical load-displacement curves of single-layer aluminum, approximately 300 μm in thickness, under quasi-static indentation loading (with a loading velocity of 3.33 × 10^−4^ m/s) are shown in Fig. 1a. The load gradually increased as the hemispherical indenter contact the plate, and the load suddenly decreased to a low level as a large catastrophic crack generated; the indenter subsequently punctured the plate. Notably, the GO-coated surface exhibited a higher penetration peak load, the dry-contact state between the indenter and plate resulted in a weaker penetrating load and a lower failure displacement. The load-displacement curves under 2.79 m/s impact loading was similar to the case of quasi-static loading as shown in Fig. 1b. All peak loads and failure points of the GO modified surface were higher than that of the dry surface under both quasi-static and the dynamic indentation conditions. Figure 1c and d revealed the superior failure load and the higher energy dissipated by the work pieces for the surface coated with the GO solution under a wide range of indentation velocities. This easily implemented GO coating method improved the energy dissipation by 15% over that of the dry-contact configuration. The finite element method (ABAQUS/Explicit software)^16^ was assisted to simulate the dynamic penetration process of single-layer configuration, the model and the material parameters were present in Supplementary Eqs S1, S2 and Table S1. Figure 2a shows that the velocity gradually decreases from the initial impact velocity to a low value, the single-layer structure undergoes elastic/plastic deformation and the velocity eventually approaches to a steady state value after the impactor penetrating the structure. The simulated curves (dot-line) agree well with the experimental curves (symbol-line) while the friction coefficient was set as 0.5^30^. Figure 2b shows the velocity gradually decreases from 2.79 m/s to a relative lower value for different friction coefficients. The residual velocity decreases from 2.46 m/s to 2.13 m/s, as shown in Fig. 2c, which corresponds to the significant increasing of absorbed energy from 4.4 J to 8.3 J as the friction coefficient *η* increases from 0 to 1. Figure 2d shows four symmetric petal-type failure mode for the contact friction coefficient equals to 0, while asymmetric petal-type with a larger plugging part presented as the friction coefficient increases to 0.5 (Fig. 2e and f). These simulation results show the contact friction coefficient is an important factor in changing the failure modes and results in improving energy dissipation property. The largest stress is concentrated at the circular centre, thus resulting in a severe centre deformation due to the direct contact position. The cross-sectional profile gradually enlarged in an arc-shaped feature as the impact time increases from 0 to 2.4 ms, this arc-shaped profile undergoes elastic recovery after the failure of the single-layer structure at 2.5 ms as shown in Fig. 2d,e and f. The stress relaxes to a lower level after the breakage and the unloading wave propagates from the failure point to the fixed boundary and then interacts with the structural vibration. The friction force is a macro equivalent force to describe the resistance between moving pairs, in real system, the friction between two solid interfaces is related with the interaction of contact state. Thus the higher energy absorption of GO smeared surface dependents on the changing of contact state. Graphene or GO is often considered as a favourable candidate for reducing friction and wear by traditional tribology tests^31, 32^. But for this transverse puncture test, the large plastic deformation and complex stress state causing the evolution of contact state is very complicated. This GO solution -coated surface seems to provide a higher contact force and the superior energy absorption. Now, the following question may arise: Why do the GO solution coated surfaces cause the structures to absorb more energy under these penetration and indentation tests? It was also worth noting in Fig. 1c and d that the peak load and the energy dissipation gradually decreased as the loading velocity increased, and then increased to a higher level under dynamic loading. What physical mechanisms dominate this improvement and govern the velocity dependency of the puncture test? Additional results were further obtained and analyzed to answer these questions.

### Contact surface states

The experimental/simulation results indicated the energy dissipation was strongly dependent on the contact conditions. Surface parameters such as the micro-asperities significantly influenced the contact morphology. The density of asperities affects the contact area, which ultimately determines the contact force^33^. Different surface conditions result in changing the true contact area between the contact pairs, thus accountings for the change in the contact frictional force. The true contact area is smaller than the calculated apparent area owing to the roughness of the surface. The sliding friction force between metal surfaces is largely dependent on the contact and subsequent fracture of micro-protrusions. From this viewpoint, the friction coefficient is proportional to the true contact area. The indenter directly contacts asperities with localized plastic deformation for the dry-contact configuration. However, the micro-valleys or grooves between micro-asperities are surrounded by the GO solution. The load was transferred by this fluid medium, thereby redistributing the load and minimizing the stress concentration. These fluid -filled micro-valleys or grooves incline to prevent the deformation of asperities, thereby affecting the micro -asperities surrounded by hydrostatic pressure. The large hydrostatic pressure resulted in a higher fracturing stress of asperities, thus possibly explaining why the viscosity of GO fluid modified layers provided a relatively high failure force and absorbed energy. This conclusion is consistent with the bio-mechanics phenomenon: the static frictional force for polypropylene and glass probes on wet skin is much greater than that on dry skin for a tangential velocity of 8 mm/s and a normal load of 2N^34^. A higher coefficient of friction of the contact surface provides superior friction force and energy absorption capacity of single-layer configurations under drop-weight transverse indentation loading.

**Figure 3:**
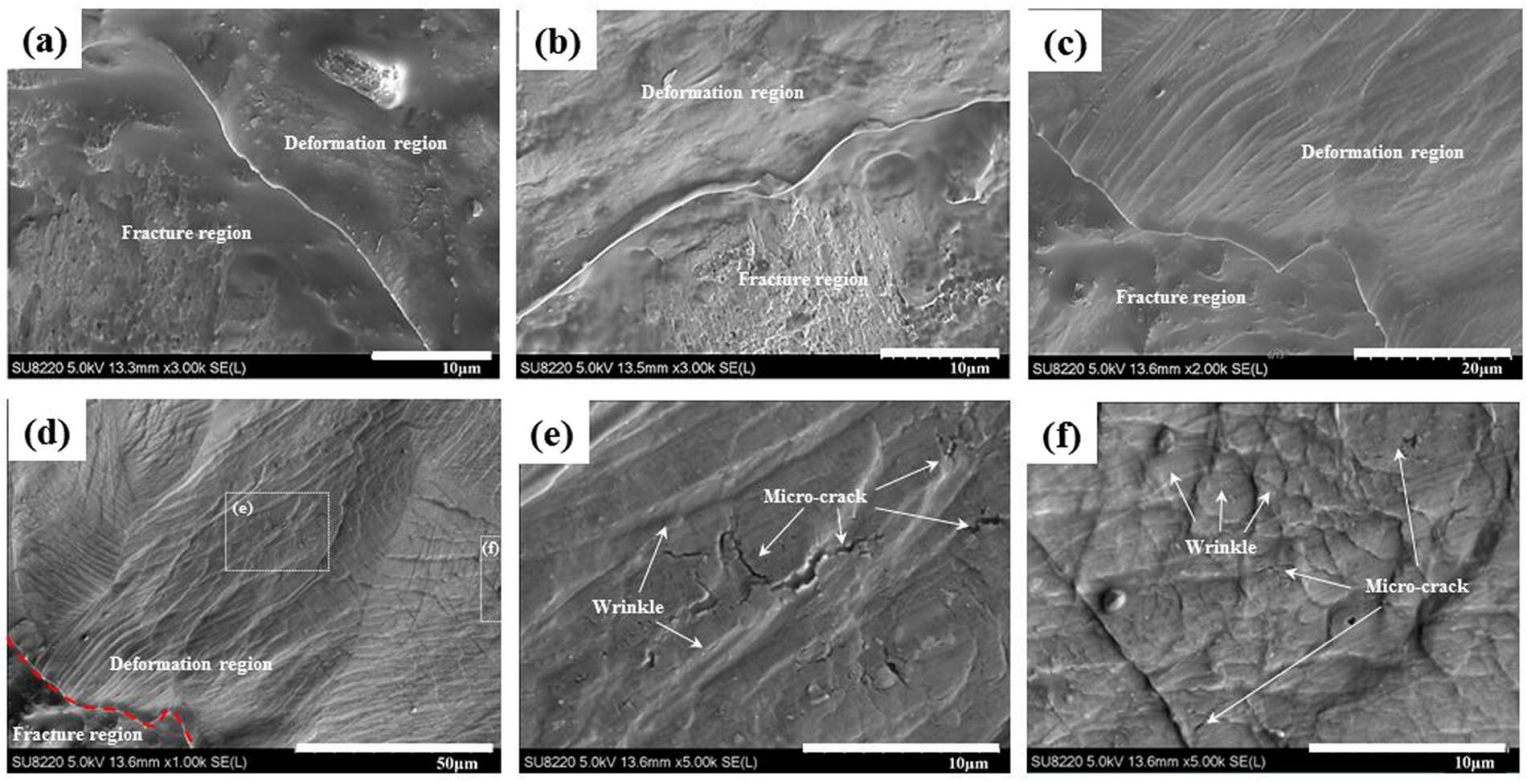
Deformation morphologies of (**a**) dry-contact surface after 3.33 × 10^−5^ m/s loading, (**b**) GO-contact surface after 3.33 × 10^−5^ m/s loading; (**c**,**d**,**e** and **f**) GO-contact after 2.79 m/s loading.

For the substrate surface infiltrated by the thin GO solution film, the quality of the contact state can be altered, thus strongly influencing the contact friction and ultimately affecting the puncture ability. The fracture/deformation junctional zone was observed to analysis the plastic deformation of contact state, as shown in Fig. 3. The deformation morphologies of the dry-contact surface seem smooth in comparison with the GO-contact surface after 3.33 × 10^−5^ m/s loading (Fig. 3a,b). The GO solution modified surface was beneficial in redistributing the load and promoting the plastic flow in the form of micro-valley and micro-hill profiles, thus possibly facilitating the evolution of contact area and inducing the uniform plastic deformation in a rough surface. Figure 3c shows the significant plastic flow after 2.79 m/s dynamic loading for GO modified surface. Numerous micro-cracks were generated among micro-wrinkles in the deformation region, as shown in Fig. 3d,e and f, thus suggesting that the stress was also well-distributed over the contact surface for the GO-solution-modified surface under dynamic loading. The structure fails at the weakest-point which induced by the primary micro-crack under lower velocity indentation. However, many micro-cracks were simultaneously generated around the primary crack due to the insufficient time to release the load during a higher velocity indentation. The evolution of the contact state was also affected by the flexibility of the substrate materials and the indenter geometry^35, 36^, but the same material and indenter geometry were used in this work.

### Grease or oil -coated surfaces

**Figure 4:**
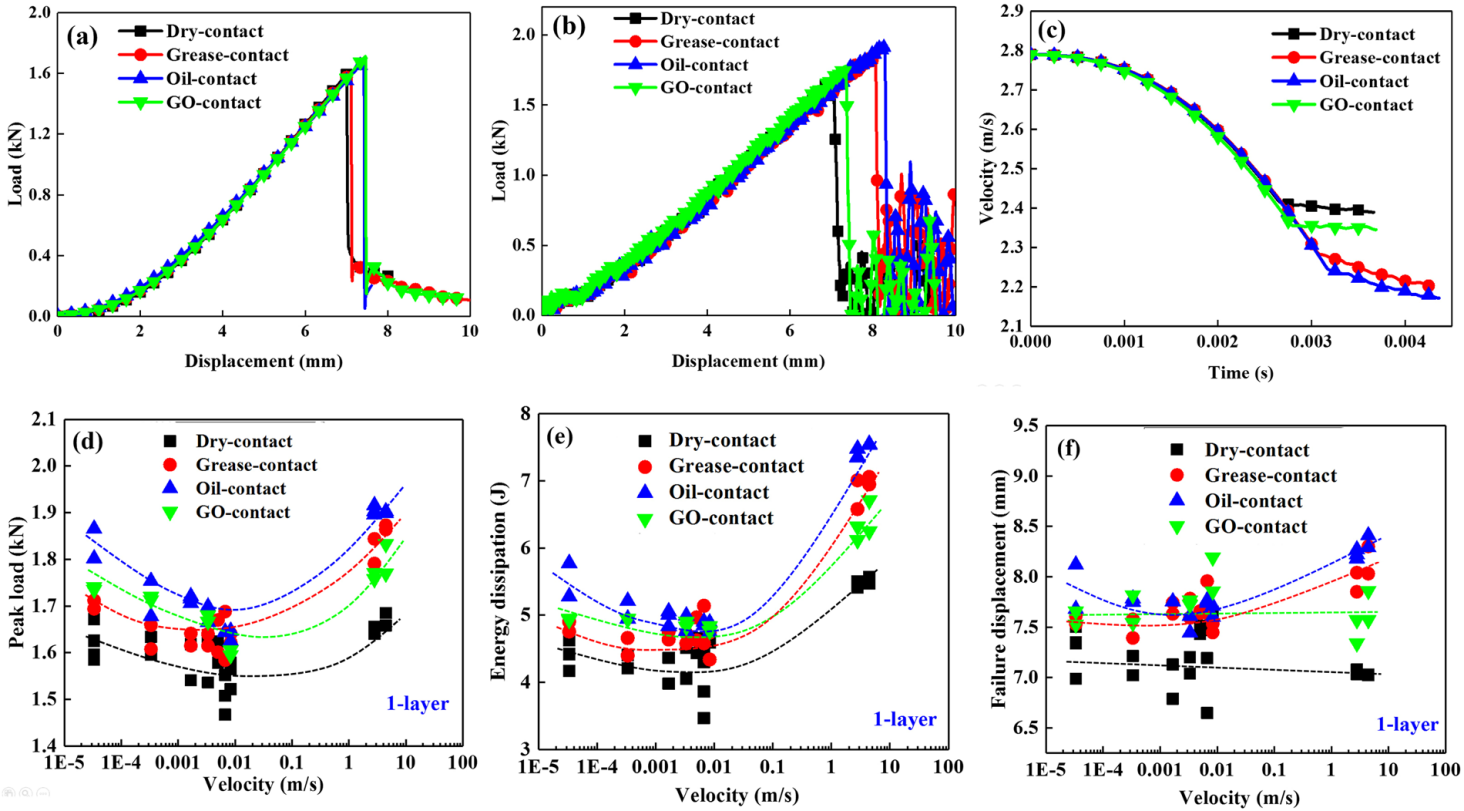
Mechanical responses of a single-layer aluminium sheet: (**a**) load-displacement curves under 3.33 × 10^−4^ m/s quasi-static loading, (**b**) load-displacement curves under 2.79 m/s impact loading, (**c**) velocity-time curves under 2.79 m/s impact loading, and mechanical properties as a function of loading velocity of (**d**) peak-load, (**e**) energy dissipation and (**f**) failure displacement.

According to the previous analysis, the state of the contact surface is not only influenced by the GO solution, but also affects by other fluid-like media. Mechanical responses under various indentation velocities were investigated for single-layer aluminium sheets with other surface modifications, such as being coated with high-vacuum grease (denoted as “grease”) or high supreme quality safe lubricant (denoted as “oil”). Figure 4a shows the weakest puncturing load and failure displacement for the dry-contact state. The oil-coated sample exhibited the highest penetration peak load, followed by the grease, GO and then dry-contact under dynamic loading, as shown in Fig. 4b. The velocity gradually decreased from 2.79 m/s as the contact time increased (Fig. 4c) and reached a stabilized lower value corresponding to the impactor passing through the sample after a 3 ms to 4 ms dynamic penetration process. The largest residual velocity was approximately 2.4 m/s for dry-contact configuration, and the oil coating showed the lowest residual velocity (2.1–2.2 m/s). The lower residual velocity of impactor also suggests higher energy dissipation, which is consistent with the simulation results (Fig. 2b and c). The detailed velocity-time curves under experimental dynamic indentation conditions for various surface modifications were provided in Supplementary Fig. S1. All the experimental data for laminar structures were summarized in Supplemental Tables S2 and S3.

**Figure 5:**
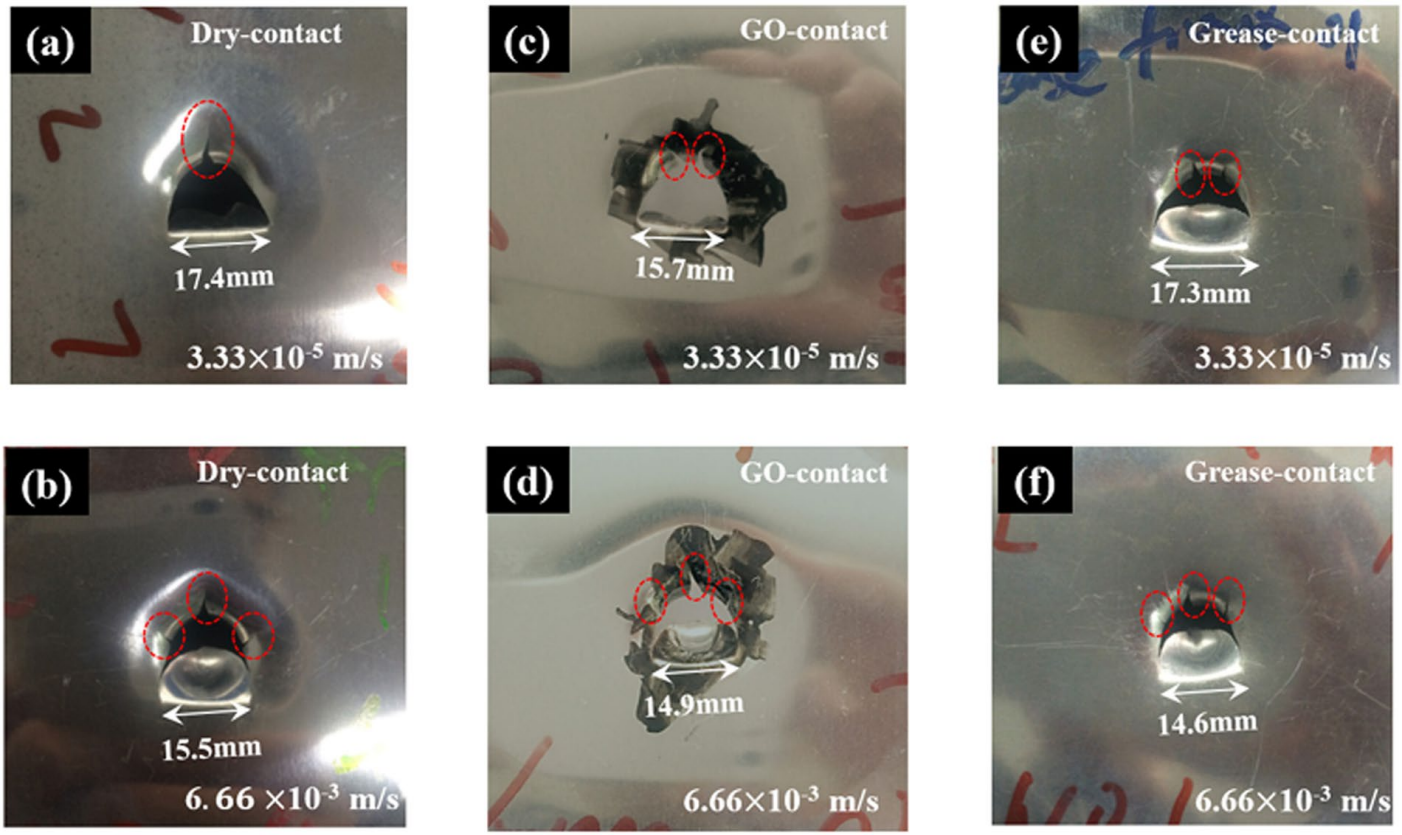
Macroscopic morphology of single-layer aluminium sheet after puncture tests: (**a**) dry-contact code under 3.33 × 10^−5^ m/s loading, (**b**) dry-contact code under 6.66 × 10^−3^ m/s loading; (**c**) GO-contact code under 3.33 × 10^−5^ m/s loading, (**d**) GO-contact code under 6.66 × 10^−3^ m/s loading; (**e**) grease-contact code under 3.33 × 10^−5^ m/s loading, (**f**) grease-contact code under 6.66 × 10^−3^ m/s loading.

Figure 4d and e reveal the adjustable modulation of loading velocity for different surface coatings; the decreasing and increasing trend is consistent with the Stribeck curve^37, 38^, which will be discussed in the following section. Figure 4f shows an increasing trend of failure displacement for the grease- or oil-modified layers, but the failure displacement for dry-contact decreased as the velocity increased. The macroscopic morphology after puncture tests were displayed in Fig. 5, the aluminum sheets reveal a larger plugging part which is similar as the simulated result in Fig. 2e. The width of the plugging part gradually decreases as the loading velocity increases for these three configurations. Meanwhile, the GO and grease coated surface present the different crack model as noted in dotted line, which reflected the alterable mode in changing of contact surface. The loading force gradually increases at the centre point, and the loading state propagates from the centre point to the boundary of the structure in the form of elastic/plastic wave; the entire structure cannot respond in time to redistribute the ever-changing load and incline in a localized manner with a smaller failure displacement under a transient dynamic penetration process.

### Layers effect

Mechanical responses of double-layers aluminium sheets under various indentation velocities were investigated to explore the layers effect. The variation tendencies of peak load and energy dissipation also started with a decreasing trend, followed by an increasing trend as the velocity increased, similarly to the results of single-layer configurations. The velocity dependency and the simulation results of double-layer configurations were detailed in Supplemental Figs S2 and S3. Notably, the grease-coated surface resulted in a lower peak load and energy dissipation level than that of the dry-contact under lower velocity loading (3.33 × 10^−4^ m/s); the grease appeared to lubricate the contact surface and decrease the friction force for this condition.

**Figure 6:**
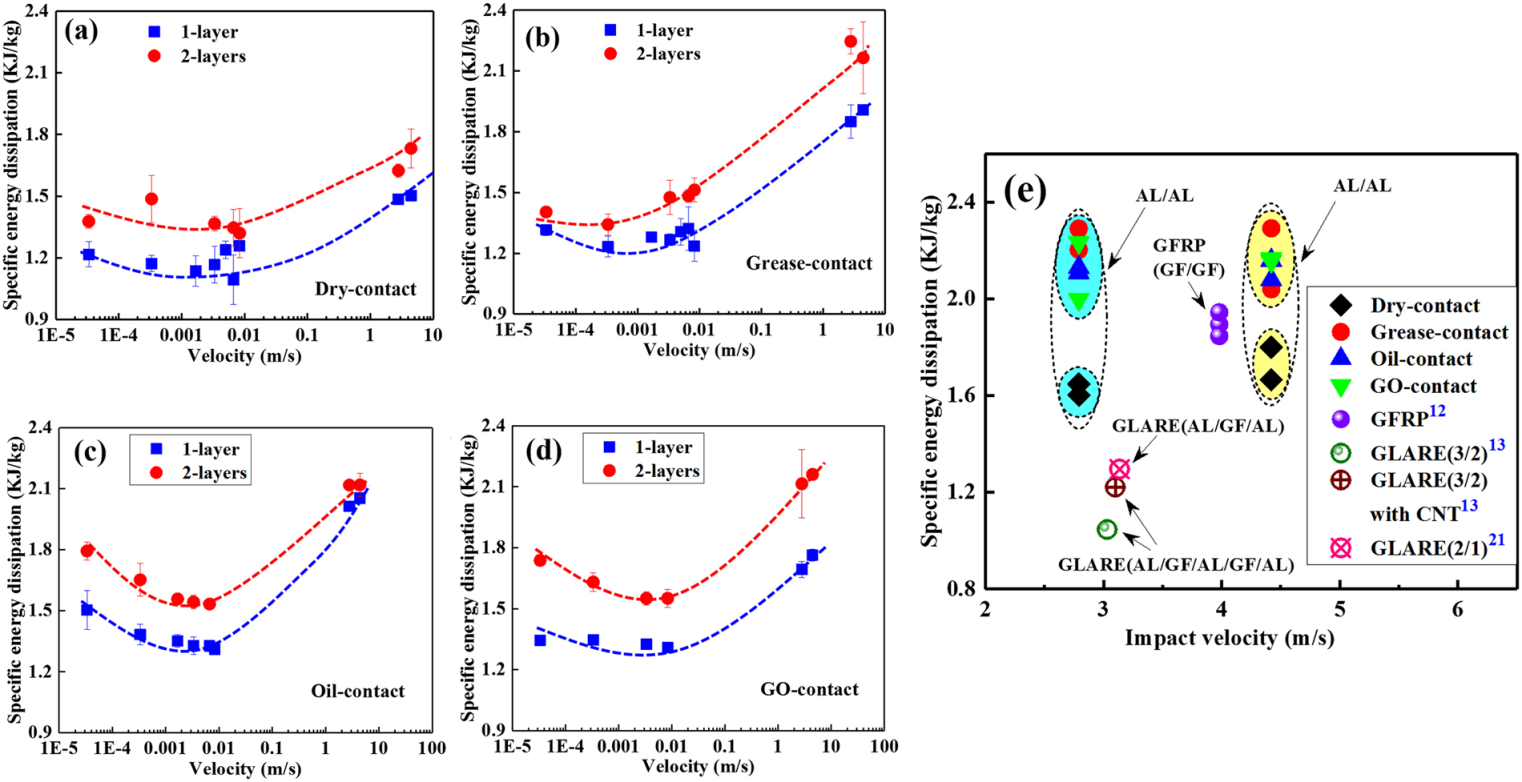
Specific energy dissipation of single and double layers for (**a**) dry-contact, (**b**) grease-contact, (**c**) oil-contact, (**d**) GO-contact and (**e**) the comparison between them.

The specific energy dissipation of the double-layer configuration were higher than that of the single layer for various contact surfaces as shown in Fig. 6. The decreasing and increasing tendency of the dry-contact sample (Fig. 6a) was more gentle than that of the grease-coated sample (Fig. 6b), which was also smoother than those of the GO- or oil-coated samples (Fig. 6c and d). The specific energy dissipation of dry-contact double-layer (Al/Al) configuration was in the range of 1.5–1.8 kJ/kg under dynamic loading, as shown in Fig. 6e, and was lower than that of the glass fabric reinforce plastic composite (GFRP-GF/GF) plate^12^ with a range from 1.8 to 2.0 kJ/kg under the similar dynamic test. For the impact-surface coated configurations, the specific energy dissipation was in the range of 1.9~2.3 kJ/kg, a value 15–40% higher than that of the dry-contact surface. These convenient surface modification methods can thus be implemented to improve puncture resistance and increase the specific energy dissipation of aluminium layers to a higher level than the GFRP composite, as shown in Fig. 6e. Besides potential difference of the intrinsic mechanical characteristic, the contact interface of impactor/GFRP configuration is considerably different from that of the impactor/aluminium configuration, and the contact state also plays an important role in influencing the energy absorption capacity of GFRP. Figure 6e also indicates that the specific energy dissipation of glass laminate aluminum reinforced epoxy (GLARE)^13, 21^ is substantially lower than that of double aluminium layers and GFRP laminates. The grease coating causes a gradually increased peak load and energy dissipation without a prominent decreasing trend in contrast to the others. These different trends suggest the potential for changeable surface conditions to provide the high-performance energy-absorption capacity.

Figure 6 shows the specific absorbed energies of double-layer configurations were 20~30% higher than that of single-layer configurations. For the double layers configuration, the interface frictional force between the two layers contributes in changing the failure mode^16^, as the back layer constrains the motion of the front layer. This additional interfacial energy and failure mode transition renders the enhanced specific energy dissipation of the double-layer configurations.

## Discussion

The lubricant solutions have been used to decrease the frictional coefficient, thus reducing abrasion and minimizing the energy loss in the operation of bearing. Previous results provided the solid evidence that applying a thin layer fluid on the impacted surface does not provide equal effectiveness in reducing the friction coefficient under transverse puncture loadings. The friction coefficient of a contact pair was regarded as a function of the load, the relative velocity and the lubricant viscosity etc., which is consistent with the classical Stribeck curve^37, 38^. The friction coefficient of a contact pair depends primarily on the geometrical interaction of micro-asperities under a lower slipping velocity and a heavy load; the friction coefficient is not sensitive to velocity under this “boundary lubrication” regime. The friction coefficient gradually decreases under a “mixed lubrication” regime. However, the load is transferred to the impactor by the fluid-film while under high-velocity “hydrodynamic lubrication”, and this regime contributes to an increasing trend as the loading velocity increases. To verify this decreasing and increasing trend, the friction coefficients as a function of velocity and load were also experimentally investigated. As shown in Supplementary Fig. S4, the friction coefficient gradually decreases as the rotation velocity increases from 50 rpm to 700 rpm under a load of 0.5N. The friction coefficient of the grease modified configuration is lower than that of the dry-contact configuration under a lower rotation velocity. However, the friction coefficient of grease modified surface is higher than that of the dry surface as the rotation velocity increases. The friction coefficient is directly proportional to the frictional force, which can be used to characterize the peak load and absorbed energy to some extent. However, the measurement of these friction coefficients lies in a wide range of time (1000 s), which is different from the dynamic transverse impact loading in instantaneous contact time. Furthermore, the dynamic puncture process of aluminium layers causes a detectable increase in temperature for the plastic work convert to the heat under transient impact loading^39, 40^, this adiabatic temperature rise decreases the viscosity of medium^41^. This instantaneously changed contact state needs more investigations in the future.

The frictional force *F*($\dot{x}$, *θ*) was regarded as a function of the sliding velocity $\dot{x}$ and the internal state variable *θ* for a rigid block pulled over a rigid plane^33^. The internal variables *θ* can be used to characterize the condition of the frictional surface, which is related to the intermediate medium, such as the GO solution, oil or grease. The internal state variable *θ* is changed by the fluid and results in an altered decreasing-increasing transition velocity. And these velocity decreasing trend give the solid evident that why the load-displacement curve under quasi-static loading is higher than that under dynamic loading for a transverse penetration loading^21^. For hydrodynamic lubrication under higher velocity, the force is transferred by the fluid film, and this regime contributes to an increasing trend as the loading velocity increases. The force between a plane and a sphere is equal to 6*πηR* ^2^ *v*_0_/*h*_0_ for a linearly viscous fluid^33^, in which the force is proportional to the dynamic viscosity of the fluid film *η* and the sliding velocity *v*_*0*_. Notably, the peak load presents different enhanced trends as the velocity increases for different coating viscosities. The contact states should firstly be taken into account in investigating the puncture resistance of protective laminar structures. The specific energy dissipation not only depends on the inherent characteristics of the materials, but also is significantly affected by the contact state for this puncture test. The results show an alterable trend of peak load and energy dissipation when the front contact surface was modified. The surface states can thus tuned by these media to meet the requirements of the protection under transverse impact loadings.

## Conclusions

The effect of a coating surface on penetration behaviour at varying velocities was investigated to elucidate the energy absorption mechanism of laminar structure under transverse loading. The application of a GO solution or high vacuum grease on the front impact surface is found to aid in increasing the puncture strength, improving the failure extension and dissipating additional energy under both quasi-static and dynamic loading conditions. The peak force/energy decrease and increase corresponded to dry-contact is more gentle than that of the fluid coated surface. The specific energy dissipation of the surface coated configurations was 15~40% higher than that of the dry-contact configuration. The simulated results agree well with the experimental results under impact velocity loadings for both single-layer and double-layer configurations. The additional interfacial frictional energy and failure mode transition of double-layer configurations result in greater specific energy dissipation than that of the single-layer configurations. The specific energy dissipation of laminar structures depends on both the inherent characteristics and the contact conditions. The surface contact states should be taken into account in investigating the impact resistance of laminar structures, and the energy absorption efficiency is expected to be improved dramatically by this convenient coating method.

## Methods

### Sample preparation

**Figure 7:**
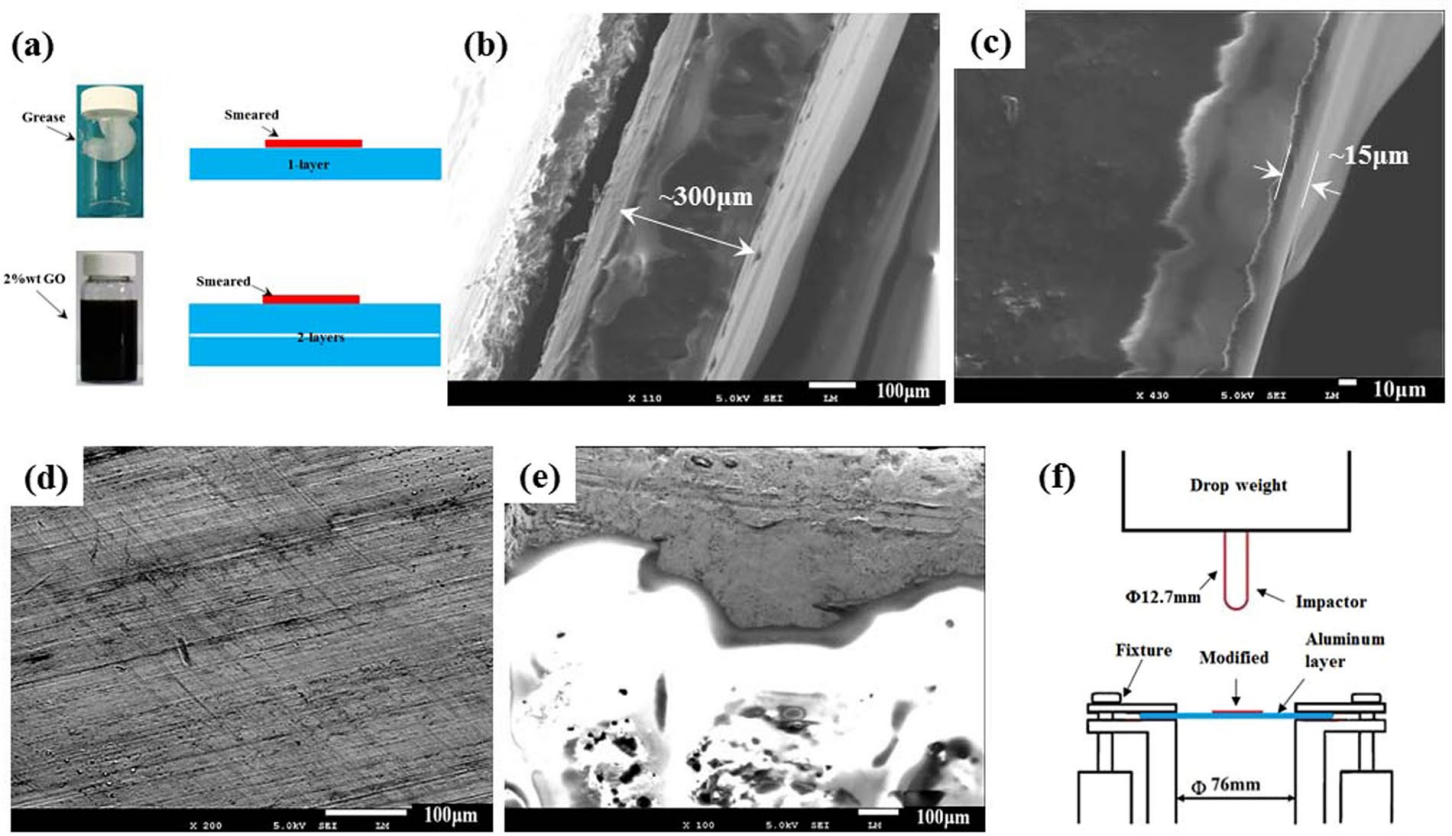
Sample and experimental configurations: (**a**) Front surface coated with high-vacuum grease or GO solution, (**b**) cross-section of the aluminium sheet, (**c**) thickness of the high-vacuum grease layer, (**d**) original front surface of the aluminium sheet, (**e**) high-vacuum grease coated front surface of the aluminium sheet, and (**f**) schematic diagram of the drop-weight impact tests.

Single-layer configurations with a thickness of 0.3 mm, as shown in Fig. 7b, were cut into 100 mm × 100 mm samples from 2024-T3 aluminum sheets. Double-layer configurations consisted of two dry -contact layers with a thickness of approximately 0.6 mm. Three types of fluid, namely, high-vacuum grease (Dow Corning Corporation, denoted as “grease”), the high -quality safe lubrication (Pennzoil Company, denoted as “oil”) and 2%*wt* GO solution were uniformly smeared on the front contact surface by using a scraper, as shown in Fig. 7a. The thickness of the high-vacuum grease thin layer was approximately 15 μm (Fig. 7c). The dry-contact front surface of the aluminium sheet and the grease-coated front surface are shown in Fig. 7d and e, respectively.

### Quasi-static and dynamic puncture test

The quasi-static indentation loading device was similar to the reference model^42^. The sample was fixed in an exposed circular hole of 76 mm, and an steel indenter with a hemispherical shaped nose and diameter of 12.7 mm was used. The loading velocity was controlled by an Instron 5569 load frame from 3.33 × 10^−5^ m/s to 8.33 × 10^−3^ m/s. The dynamic drop-weight loading device was similar to the ref. 16. As shown in Fig. 7f, the configuration of the fixed sample and impactor nose were identical to the quasi-static indentation test configuration. The initial impact velocity of the impactor with mass of 5.131 kg was set to 2.79 m/s and 4.42 m/s. The samples with each test configuration were repeated for 2~4 times to obtain the reliable conclusion. The friction coefficient was determined using a universal tribometer (Mod. UMT-3MT) with a Φ4 mm steel-ball on a controlled rotating disc under the normal loads ranging from 0.1 N to 0.5 N at the speeds ranging from 50 to 1000 rpm. The microstructure morphology was observed with a field emission scanning electron microscope (FESEM, JEOL, JSM-6700F; HITACHI UHR FE-SEM SU8220).

## Electronic supplementary material

Supplementary information
