# Supplementary material for: Modification of the contact surfaces for improving the puncture resistance of laminar structures
Source: Sci Rep. 2017 Jul 26;7:6615. doi: 10.1038/s41598-017-06007-3 (PMC5529432; doi:10.1038/s41598-017-06007-3)
Supplement: Supplementary file 1 — Supplementary information [file 41598_2017_6007_MOESM1_ESM.doc]

**Modification of the contact surfaces for improving the** **puncture resistance of laminar structures**

Pengfei Wang, Jinglei Yang, Xin Li, Mao Liu, Xin Zhang, Dawei Sun, Chenlu Bao, Guangfa Gao, Mohd Yazid Yahya, Songlin Xu

**Simulation method**

ABAQUS/Explicit finite element method was used to study the dynamic puncture behavior of single and double-aluminum layers subjected to drop-weight impact. A hemispherical nose-shaped steel impactor with diameter 12.7mm and mass 5.131kg was defined as discrete rigid body. The center contact region was assigned by more seeds for accurately simulating the penetrating process of structure. The contact pairs were arranged as the penalty formulation, and the values of friction coefficient from 0.0 to 1.0 were assigned. The surrounding edge of aluminum sheets are all clamped.

The aluminum sheet was set as Johnson-Cook model ^1,2^ as expressed in Eq. (S1).

(S1)

where $\sigma$ represents flow stress, $\varepsilon$ represents plastic strain, is normalized strain rate. *n* is the strain hardening exponent, and *A*, *B* and *C* are parameters. Meanwhile, Johnson-Cook damage criterion is adopted to observe the dynamic damage process of aluminum sheets. The equivalent fracture strain $\varepsilon_{f}$as expressed in Eq. (S2).

(S2)

where $\sigma^{*}$is the stress triaxiality ratio. $D_{1}$, $D_{2}$, $D_{3}$, $D_{4}$ and $D_{5}$are material parameters. The details of the material parameters are shown in Table S1.

**Table S1.** Material parameters of Al-alloy ^3,4^.

| Material | A(MPa) | B(MPa) | n | C | m | D_1_ | D_2_ | D_3_ | D_4_ | D_5_ |
| --- | --- | --- | --- | --- | --- | --- | --- | --- | --- | --- |
| 2024-T3 | 369 | 684 | 0.73 | 0.0083 | 1.7 | 0.13 | 0.13 | 1.5 | 0.011 | 0 |

**
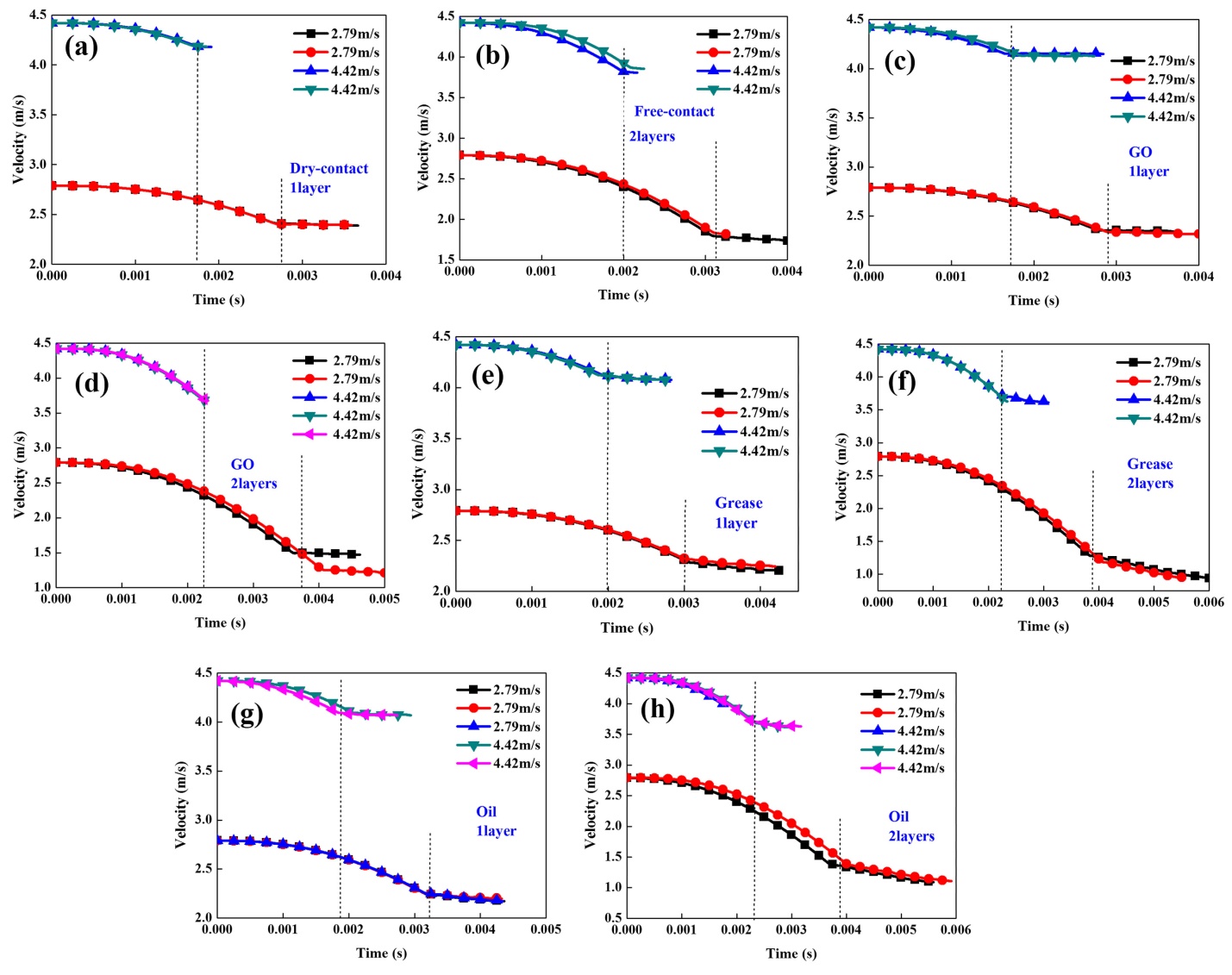
**

**Fig. S1.** Velocity-time curves under dynamic loadings. (a) free-contact configuration, single-layer; (b) free-contact configuration, double-layers; (c) GO configuration, single-layer; (d) GO configuration, double-layers; (e) grease configuration, single-layer; (f) grease configuration, double-layers; (g) grease configuration, single-layer; (h) grease configuration, double-layers.


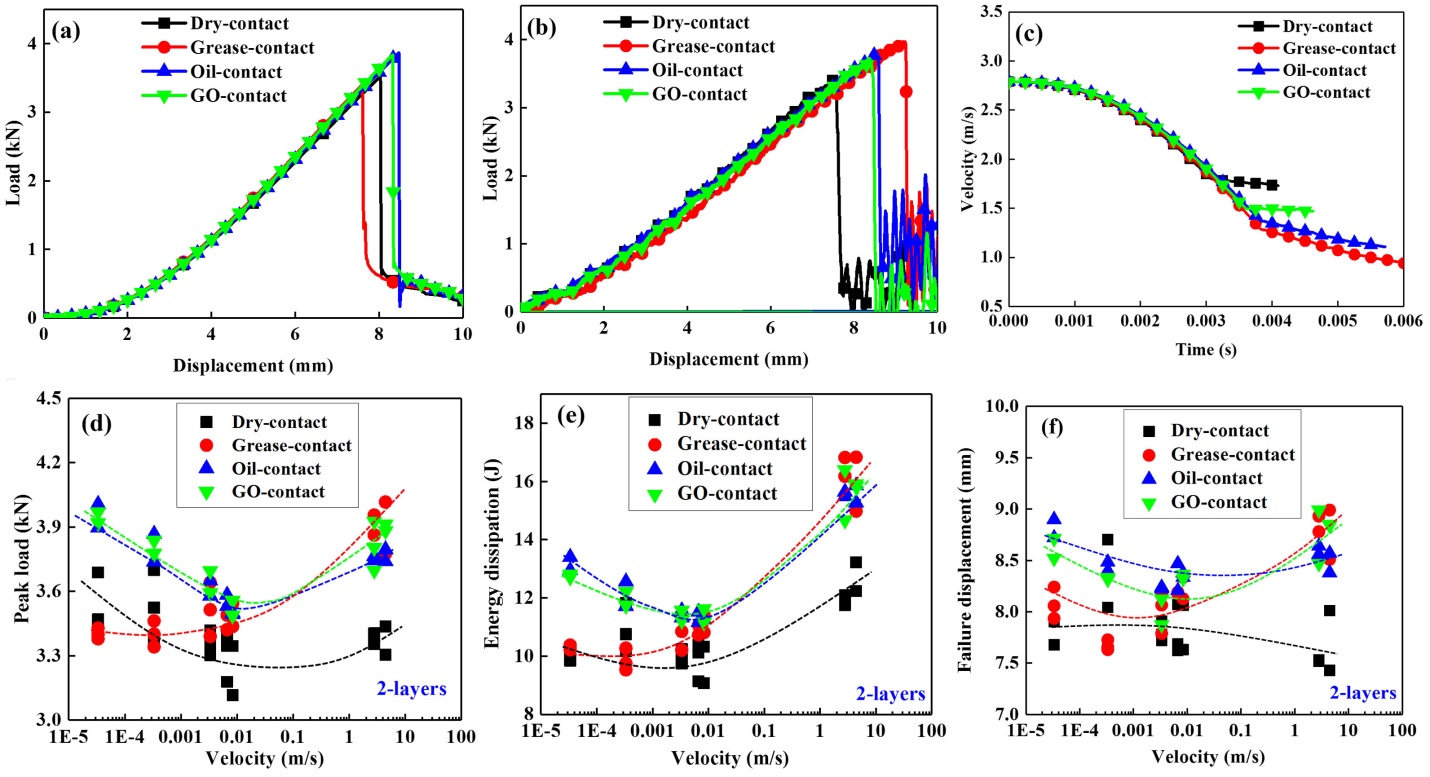


**Fig. S2. Mechanical properties of double-layers aluminum sheets** **as a function of loading velocity:** (a) peak-load, (b) energy dissipation and (c) failure displacement, and mechanical properties as a function of loading velocity of (d) peak-load, (e) energy dissipation and (f) failure displacement.


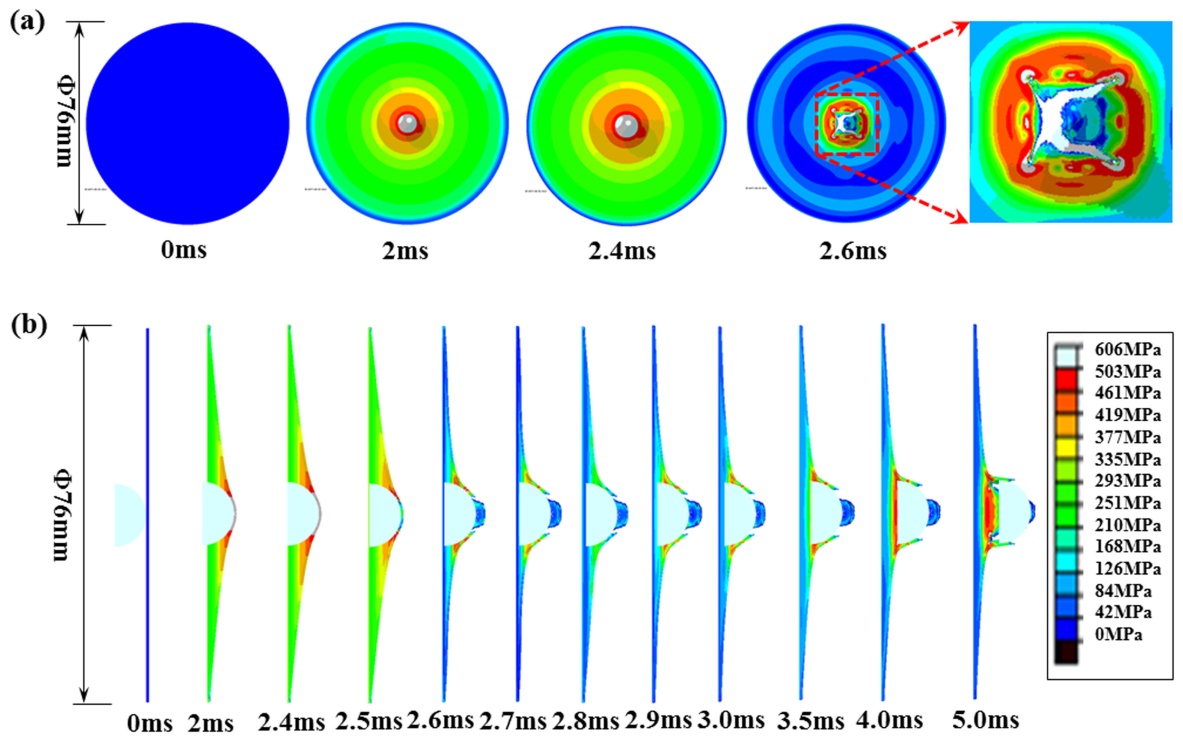


**Fig.S3. Simulated dynamic deformation process of double-layer aluminum (friction coefficient η=0.5) under 2.79m/s impact loading** (a) rear view and (b) cross-sectional view.


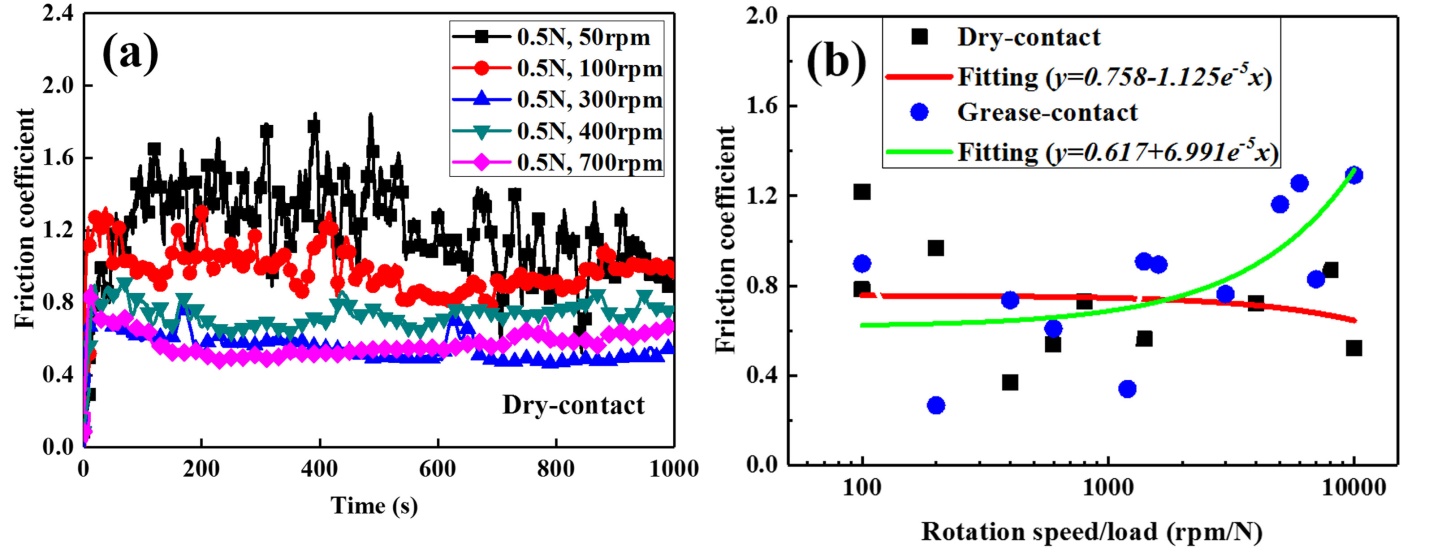


**Fig. S4**. Friction coefficient as a function of rotation velocity and load

**Table S2.**Experimental data of different surface modification for Single-aluminum layer.

| Surface state | | Loading velocity (m/s) | Peak load (kN) | Failure disp. (mm) | Energy dissipation (J) | Specific energy dissipation (KJ/kg) |
| --- | --- | --- | --- | --- | --- | --- |
| Dry-contact | | 3.33×10^-5^ | 1.625 | 7.348 | 4.419 | 1.217±0.062 |
|  |  |  | 1.585 | 6.986 | 4.170 |  |
|  |  |  | 1.670 | 7.336 | 4.623 |  |
|  |  |  | 1.680 | 7.501 | 4.667 |  |
|  |  | 3.33×10^-4^ | 1.596 | 7.021 | 4.206 | 1.172±0.039 |
|  |  |  | 1.634 | 7.212 | 4.406 |  |
|  |  | 1.67×10^-3^ | 1.628 | 7.128 | 4.366 | 1.136±0.074 |
|  |  |  | 1.541 | 6.787 | 3.981 |  |
|  |  | 3.33×10^-3^ | 1.536 | 7.039 | 4.054 | 1.167±0.089 |
|  |  |  | 1.621 | 7.201 | 4.517 |  |
|  |  | 5×10^-3^ | 1.624 | 7.515 | 4.659 | 1.238±0.043 |
|  |  |  | 1.578 | 7.429 | 4.434 |  |
|  |  | 6.67×10^-3^ | 1.374 | 3.466 | 6.647 | 1.093±0.120 |
|  |  |  | 1.467 | 3.861 | 7.191 |  |
|  |  |  | 1.522 | 4.438 | 7.470 |  |
|  |  |  | 1.508 | 4.300 | 7.493 |  |
|  |  | 8.33×10^-3^ | 1.579 | 7.580 | 4.651 | 1.258±0.012 |
|  |  |  | 1.565 | 7.654 | 4.590 |  |
|  |  | 2.79 | 1.640 | 7.03 | 5.41 | 1.485±0.017 |
|  |  |  | 1.655 | 7.08 | 5.50 |  |
|  |  | 4.42 | 1.685 | 7.03 | 5.57 | 1.503±0.019 |
|  |  |  | 1.658 | 7.02 | 5.47 |  |
| GO-contact | 3.33×10^-5^ | | 1.741 | 7.658 | 4.947 | 1.345±0.003 |
|  |  |  | 1.735 | 7.526 | 4.932 |  |
|  | 3.33×10^-4^ | | 1.713 | 7.817 | 4.940 | 1.346±0.002 |
|  |  |  | 1.720 | 7.542 | 4.947 |  |
|  | 3.33×10^-3^ | | 1.680 | 7.723 | 4.903 | 1.325±0.014 |
|  |  |  | 1.667 | 7.766 | 4.829 |  |
|  | 8.33×10^-3^ | | 1.606 | 8.192 | 4.837 | 1.308±0.013 |
|  |  |  | 1.595 | 7.856 | 4.768 |  |
|  | 2.79 | | 1.758 | 7.34 | 6.12 | 1.693±0.039 |
|  |  |  | 1.771 | 7.57 | 6.32 |  |
|  | 4.42 | | 1.770 | 7.57 | 6.25 | 1.764±0.089 |
|  |  |  | 1.833 | 7.86 | 6.71 |  |
| Grease-contact | 3.33×10^-5^ | | 1.694 | 7.529 | 4.756 | 1.315±0.028 |
|  |  |  | 1.712 | 7.610 | 4.902 |  |
|  | 3.33×10^-4^ | | 1.608 | 7.392 | 4.398 | 1.233±0.051 |
|  |  |  | 1.659 | 7.577 | 4.661 |  |
|  | 1.67×10^-3^ | | 1.615 | 7.631 | 4.637 | 1.279±0.023 |
|  |  |  | 1.641 | 7.743 | 4.755 |  |
|  | 3.33×10^-3^ | | 1.640 | 7.783 | 4.727 | 1.267±0.028 |
|  |  |  | 1.615 | 7.592 | 4.579 |  |
|  | 5×10^-3^ | | 1.670 | 7.658 | 4.966 | 1.306±0.066 |
|  |  |  | 1.601 | 7.639 | 4.625 |  |
|  | 6.67×10^-3^ | | 1.688 | 7.956 | 5.139 | 1.323±0.107 |
|  |  |  | 1.584 | 7.639 | 4.581 |  |
|  | 8.33×10^-3^ | | 1.659 | 7.533 | 4.739 | 1.236±0.077 |
|  |  |  | 1.608 | 7.446 | 4.340 |  |
|  | 2.79 | | 1.844 | 8.04 | 7.01 | 1.850±0.083 |
|  |  |  | 1.791 | 7.85 | 6.58 |  |
|  | 4.42 | | 1.873 | 8.30 | 7.06 | 1.907±0.021 |
|  |  |  | 1.864 | 8.03 | 6.95 |  |
| Oil-contact | 3.33×10^-5^ | | 1.802 | 7.680 | 5.278 | 1.504±0.095 |
|  |  |  | 1.866 | 8.120 | 5.774 |  |
|  | 3.33×10^-4^ | | 1.678 | 7.783 | 4.948 | 1.383±0.013 |
|  |  |  | 1.754 | 7.749 | 5.212 |  |
|  | 1.67×10^-3^ | | 1.721 | 7.754 | 5.054 | 1.352±0.031 |
|  |  |  | 1.716 | 7.752 | 5.004 |  |
|  |  |  | 1.706 | 6.536 | 4.835 |  |
|  | 3.33×10^-3^ | | 1.665 | 7.445 | 4.766 | 1.328±0.043 |
|  |  |  | 1.699 | 7.610 | 4.991 |  |
|  | 6.67×10^-3^ | | 1.644 | 7.774 | 4.901 | 1.327±0.011 |
|  |  |  | 1.645 | 7.766 | 4.846 |  |
|  | 8.33×10^-3^ | | 1.647 | 7.709 | 4.888 | 1.311±0.028 |
|  |  |  | 1.625 | 7.602 | 4.744 |  |
|  | 2.79 | | 1.916 | 8.27 | 7.48 | 2.014±0.020 |
|  |  |  | 1.896 | 8.22 | 7.36 |  |
|  |  |  | 1.903 | 8.18 | 7.35 |  |
|  | 4.42 | | 1.903 | 8.41 | 7.54 | 2.054±0.002 |
|  |  |  | 1.899 | 8.29 | 7.55 |  |

**Table S3.**Experimental data of different surface modification for double-aluminum layer.

| Surface state | | Loading velocity (m/s) | Peak load (kN) | Failure disp. (mm) | Energy dissipation (J) | Specific energy dissipation (KJ/kg) |
| --- | --- | --- | --- | --- | --- | --- |
| Pristine-contact | | 3.33×10^-5^ | 3.470 | 7.902 | 10.313 | 1.377±0.033 |
|  |  |  | 3.437 | 7.912 | 10.178 |  |
|  |  |  | 3.389 | 7.679 | 9.848 |  |
|  |  | 3.33×10^-4^ | 3.699 | 8.702 | 11.830 | 1.486±0.115 |
|  |  |  | 3.374 | 7.690 | 10.162 |  |
|  |  |  | 3.524 | 8.042 | 10.757 |  |
|  |  | 3.33×10^-3^ | 3.302 | 7.911 | 9.746 | 1.366±0.035 |
|  |  |  | 3.358 | 7.780 | 10.111 |  |
|  |  |  | 3.417 | 7.720 | 10.245 |  |
|  |  | 6.67×10^-3^ | 3.178 | 7.624 | 9.140 | 1.346±0.090 |
|  |  |  | 3.395 | 8.070 | 10.395 |  |
|  |  |  | 3.346 | 7.686 | 10.119 |  |
|  |  | 8.33×10^-3^ | 3.345 | 8.061 | 10.320 | 1.320±0.120 |
|  |  |  | 3.116 | 7.632 | 9.069 |  |
|  |  | 2.79 | 3.404 | 7.53 | 12.10 | 1.624±0.033 |
|  |  |  | 3.353 | 7.52 | 11.76 |  |
|  |  | 4.42 | 3.304 | 8.01 | 13.22 | 1.732±0.095 |
|  |  |  | 3.436 | 7.43 | 12.23 |  |
| GO-contact | 3.33×10^-5^ | | 3.969 | 8.713 | 12.810 | 1.738±0.0094 |
|  |  |  | 3.919 | 8.521 | 12.718 |  |
|  | 3.33×10^-4^ | | 3.837 | 8.333 | 12.213 | 1.631±0.045 |
|  |  |  | 3.776 | 8.316 | 11.745 |  |
|  | 3.33×10^-3^ | | 3.593 | 8.123 | 11.225 | 1.551±0.033 |
|  |  |  | 3.696 | 7.873 | 11.570 |  |
|  | 8.33×10^-3^ | | 3.556 | 8.363 | 11.628 | 1.551±0.045 |
|  |  |  | 3.488 | 8.322 | 11.162 |  |
|  | 2.79 | | 3.698 | 8.47 | 16.40 | 2.115±0.167 |
|  |  |  | 3.926 | 8.99 | 14.67 |  |
|  | 4.42 | | 3.912 | 8.85 | 15.83 | 2.161±0.008 |
|  |  |  | 3.882 | 8.84 | 15.91 |  |
| Grease-contact | 3.33×10^-5^ | | 3.428 | 7.935 | 10.378 | 1.404±0.011 |
|  |  |  | 3.339 | 8.241 | 10.220 |  |
|  |  |  | 3.379 | 8.058 | 10.337 |  |
|  | 3.33×10^-4^ | | 3.400 | 7.631 | 9.754 | 1.342±0.052 |
|  |  |  | 3.462 | 7.727 | 10.277 |  |
|  |  |  | 3.371 | 7.657 | 9.530 |  |
|  | 3.33×10^-3^ | | 3.390 | 7.789 | 10.205 | 1.476±0.085 |
|  |  |  | 3.641 | 8.065 | 11.459 |  |
|  |  |  | 3.514 | 8.178 | 10.850 |  |
|  | 6.67×10^-3^ | | 3.490 | 8.174 | 11.063 | 1.483±0.032 |
|  |  |  | 3.421 | 8.191 | 10.731 |  |
|  | 8.33×10^-3^ | | 3.436 | 8.127 | 10.810 | 1.513±0.059 |
|  |  |  | 3.543 | 8.334 | 11.424 |  |
|  | 2.79 | | 3.862 | 8.78 | 16.18 | 2.246±0.062 |
|  |  |  | 3.955 | 8.93 | 16.82 |  |
|  | 4.42 | | 3.775 | 8.51 | 14.98 | 2.165±0.178 |
|  |  |  | 4.016 | 8.99 | 16.83 |  |
| Oil-contact | 3.33×10^-5^ | | 3.896 | 8.898 | 12.950 | 1.794±0.044 |
|  |  |  | 4.006 | 8.721 | 13.403 |  |
|  | 3.33×10^-4^ | | 3.867 | 8.483 | 12.560 | 1.652±0.081 |
|  |  |  | 3.738 | 8.400 | 11.716 |  |
|  | 3.33×10^-3^ | | 3.649 | 8.225 | 11.570 | 1.558±0.024 |
|  |  |  | 3.578 | 8.239 | 11.324 |  |
|  | 6.67×10^-3^ | | 3.530 | 8.206 | 11.158 | 1.543±0.034 |
|  |  |  | 3.580 | 8.468 | 11.516 |  |
|  | 8.33×10^-3^ | | 3.492 | 8.379 | 11.260 | 1.533±0.001 |
|  |  |  | 3.500 | 8.335 | 11.253 |  |
|  | 2.79 | | 3.763 | 8.56 | 15.48 | 2.118±0.015 |
|  |  |  | 3.743 | 8.64 | 15.64 |  |
|  | 4.42 | | 3.740 | 8.57 | 15.86 | 2.119±0.057 |
|  |  |  | 3.795 | 8.38 | 15.27 |  |

**Reference:**

1 Johnson, G.R. and Cook, W.H. A constitutive model and data for metals subjected to large strains, high strain rates and high temperatures. *Proc. of the seventh international symposium on ballistics*, The Hague, Netherland, pp. 541-547 (1983).

2 Johnson, G.R. and Cook, W.H. Fracture characteristics of three metals subjected to various strains, strain rates, temperatures and pressures. *Eng. Fract. Mech* **21**, 31-48 (1985).

3 Lesuer, D. Experimental investigations of material models for Ti-6AL4V and 2024-T3. *US Department of Energy* (1999).

4 Zhu, S. and Chai, G.B. Low-velocity impact response of fibre-metal laminates – Experimental and finite element analysis. *Compos. Sci. Technol.* **72**, 1793-1802 (2012).
